# Feeling of Pleasure to High-Intensity Interval Exercise Is Dependent of the Number of Work Bouts and Physical Activity Status

**DOI:** 10.1371/journal.pone.0152752

**Published:** 2016-03-30

**Authors:** Danniel Thiago Frazão, Luiz Fernando de Farias Junior, Teresa Cristina Batista Dantas, Kleverton Krinski, Hassan Mohamed Elsangedy, Jonato Prestes, Sarah J. Hardcastle, Eduardo Caldas Costa

**Affiliations:** 1 Department of Physical Education, Federal University of Rio Grande do Norte, Natal, RN, Brazil; 2 Department of Physical Education, Federal University of Vale do São Francisco, Petrolina, PE, Brazil; 3 Graduation Program on Physical Education, Catholic University of Brasilia, Brasilia, DF, Brazil; 4 School of Psychology and Speech Pathology, Curtin University, Perth, Australia; 5 Postgraduate Program in Health Sciences, Federal University of Rio Grande do Norte, Natal, RN, Brazil; University of Rome, ITALY

## Abstract

**Objectives:**

To examine the affective responses during a single bout of a low-volume HIIE in active and insufficiently active men.

**Materials and methods:**

Fifty-eight men (aged 25.3 ± 3.6 years) volunteered to participate in this study: i) active (n = 29) and ii) insufficiently active (n = 29). Each subject undertook i) initial screening and physical evaluation, ii) maximal exercise test, and iii) a single bout of a low-volume HIIE. The HIIE protocol consisted of 10 x 60s work bouts at 90% of maximal treadmill velocity (MTV) interspersed with 60s of active recovery at 30% of MTV. Affective responses (Feeling Scale, -5/+5), rating of perceived exertion (Borg’s RPE, 6–20), and heart rate (HR) were recorded during the last 10s of each work bout. A two-factor mixed-model repeated measures ANOVA, independent-samples t test, and chi-squared test were used to data analysis.

**Results:**

There were similar positive affective responses to the first three work bouts between insufficiently active and active men (p > 0.05). However, insufficiently active group displayed lower affective responses over time (work bout 4 to 10) than the active group (p < 0.01). Also, the insufficiently active group displayed lower values of mean, lowest, and highest affective response, as well as lower values of affective response at the highest RPE than the active group (p < 0.001). There were no differences in the RPE and HR between the groups (p > 0.05).

**Conclusions:**

Insufficiently active and active men report feelings of pleasure to few work bouts (i.e., 3–4) during low-volume HIIE, while the affective responses become more unpleasant over time for insufficiently active subjects. Investigations on the effects of low-volume HIIE protocols including a fewer number of work bouts on health status and fitness of less active subjects would be interesting, especially in the first training weeks.

## Introduction

It is well established that ‘all out’ high-intensity interval training (HIIT) results in a host of physiological adaptations including improvements in health and fitness [1–3]. In addition, these improvements have been reported to be equal or superior to traditional continuous aerobic training, while HIIT involves a substantially lower total training volume [4,5]. However, ‘all out’ HIIT requires specialized equipment [6] and a high level of motivation, and may not be safe, tolerable or practical for a largely sedentary population [7,8]. In this sense, Hardcastle et al. [7] has advocated that ‘all out’ HIIT or SIT (sprint interval training) is unlikely to be taken up by the majority of the sedentary population because these training modalities are likely to evoke a high degree of negative affect, which may lead to an avoidant response with the prospect of participation in future sessions.

The high levels of exertion induced by ‘all out’ HIIT or SIT have led investigators to study the impact of less strenuous and more practical and feasible HIIT protocols [8–11]. Gibala et al. [12] proposed a low-volume HIIT protocol consisting of 10 x 60s work bouts at ~90% of maximal heart rate (HRmax), interspersed with 60s of recovery. This low-volume HIIT model improves aerobic capacity and increases mitochondrial enzyme content and activity, resulting in enhanced muscle oxidative potential [10,11]. Thus, low-volume HIIT likely represents a useful strategy to enhance whole body physiological function and prevent cardiometabolic diseases [13]. Moreover, considering that ‘lack of time’ is the most commonly cited barrier to regular exercise participation [14,15], low-volume HIIT could be an effective strategy to improve health and fitness.

Clearly, there is a strong necessity to develop time-efficient exercise strategies (e.g., low-volume HIIT) to improve health and fitness of sedentary and insufficiently active subjects and maintain these benefits in active subjects. However, these time-efficient strategies should not be perceived as aversive [7]. The American College of Sports Medicine (ACSM) exercise guidelines state that exercise-induced feelings of fatigue and negative affect can act as a deterrent to continued participation [16]. On the other hand, feeling of pleasure during exercise is a determinant of physical activity participation and adherence, as previously reported in observational studies [17]. Thus, strategies to enhance the likelihood of gaining pleasurable feelings are likely to contribute to exercise maintenance and subsequent benefits to health and fitness.

Considering that exercise induced feelings of pleasure and positive affect from a single bout of exercise predicts physical activity participation and adherence [17], it is important to study the affective responses to a single bout of high-intensity interval exercise (HIIE) in subjects of varying physical activity status. Previous studies have shown that active subjects report more positive affective response (feeling of pleasure) than sedentary subjects during exercise prescribed from moderate to high intensities [18–22]. However, these studies compared the affective response between sedentary and active subjects during continuous exercise protocols. To the best of our knowledge, no study has yet compared the affective response during a single bout of low-volume HIIE between subjects with different physical activity status (i.e., sufficiently physically active and insufficiently physically active). This is an important research gap related to the field of exercise prescription for health promotion.

Furthermore, there is a gap in ecological investigations that explore affective responses to low-volume HIIE involving subjects of varying physical activity status in a ‘real world’ environment using readily available equipment and simple tools to measure affective states that are likely to influence exercise adherence [7]. Therefore, this investigation aimed to examine the affective responses (i.e., feeling of pleasure/displeasure) during a single bout of a low-volume and practical HIIE protocol in active and insufficiently active men. Our initial hypothesis was that active subjects would display a more pleasurable response to a single bout of low-volume HIIE compared to their insufficiently active counterparts.

## Materials and Methods

### Study design

This is a single occasion trial with repeated measures across two groups designed to compare the affective responses during a single bout of HIIE in subjects with different physical activity status. Subjects were separated into two groups according to their physical activity status: i) active (n = 29) and ii) insufficiently active (n = 29). Each subject undertook the following procedures: i) initial screening and physical evaluation; ii) maximal exercise test; iii) a single bout of a low-volume HIIE protocol. The subjects performed the maximal exercise test and the HIIE bout with an interval of one week. Initially, the subjects were screened for medical history and physical activity readiness. In the same day, body weight (kg) and height (m) were measured. Body mass index was calculated as weight (kg) divided by the square of height in meters (kg/m^2^). Lastly, subjects were asked to avoid vigorous physical activity, caffeinated products, and alcohol consumption 24h before the maximal exercise test and the HIIE bout, and to maintain a good sleeping pattern and normal dietary habits. This study was approved by the University Human Research Ethics Committee (CAAE: 28710414.1.0000.5537).

### Participants

Fifty-eight men (aged 25.3 ± 3.6 years) volunteered to participate of this study. Subjects were recruited via personal or printed invitations in the university setting as well as via e-mail and online social networks. All subjects completed a medical history questionnaire and the physical activity readiness questionnaire (PAR-Q) before the study. Inclusion criteria were: i) be classified as apparently healthy; ii) without any contraindications to exercise; iii) injury-free at the time of this study. Exclusion criteria were as follows: i) one positive response on the PAR-Q, ii) body mass index (BMI) < 18.5 kg^.^m^2^ or BMI > 30.0 kg^.^m^2^, iii) being a smoker or recently quitting smoking (in the previous 6 months) were exclusion criteria, and iv) diagnosis of cardiovascular, metabolic, and orthopedic disease or any other contraindications for physical activity, as determined by a medical history. Subjects were informed about all procedures of the study, and gave written informed consent.

### Procedures

#### Physical activity level assessment

The classification of the physical activity status was based on the ACSM guidelines [16] using the short-version of the International Physical Activity Questionnaire (IPAQ) [23,24]. The insufficiently active group included subjects that performed less than 150 min^.^wk^-1^ of moderate physical activity and/or less than 75 min^.^wk^-1^ of vigorous physical activity during the last three months, while the active group met at least one or both the above mentioned criteria. Although the IPAQ only refers to physical activity participation in the previous week, subjects were also asked whether the pattern of physical activity reported in the IPAQ was consistent with the previous three months. Only subjects that reported a consistent pattern of physical activity in the last three months were included in the study. The IPAQ has test-retest reliability (Spearman’s rho = 0.8) and criterion validity (against the MTI accelerometer), which is comparable to most self-report validation studies [25].

Subjects from the active group habitually performed exercise including non-competitive sports, resistance training, aerobic exercises, and were familiar with interval training, while those from the insufficiently active group were not involved in any regular physical activity and/or exercise program. However, none of the participants (across both groups) had previous experience with the HIIE protocol used in the present study.

#### Maximal exercise test

Subjects performed a maximal exercise test to determine the maximal treadmill velocity (MTV) and heart rate (HRmax). All subjects had previous experience in exercising on a treadmill, even the insufficiently active men. Initially, the warm-up consisted of walking at 4 km^.^h^-1^ for five minutes on a motorized treadmill (Inbrasport^®^, Porto Alegre, BRA). For the insufficiently active group, the incremental test began at 4 km^.^h^-1^ with 1% of inclination for 1-min followed by fixed increments of 1 km^.^h^-1^ per minute until volitional exhaustion. For the active group, the test began at 6 km^.^h^-1^ with 1% of inclination for 1-min followed by fixed increments of 1 km^.^h^-1^ per minute until the volitional exhaustion. The MTV was defined as the velocity achieved during the last full stage before volitional exhaustion. HR (beats/minute) was continuously recorded throughout the test using a Polar Monitoring System (Polar Electro^®^, Oy, Kempele, Finland). All subjects achieved ≥ 95% of age-predicted maximal HR (220 –age) at the moment of volitional exhaustion. The Borg’s RPE scale [26] was used to assess whole body perceived exertion during each stage of the maximal exercise test. All participants received standardized instructions on the use of Borg’s RPE Scale before the test [27]. The tests were performed between 8–12 and 8–14 minutes for the insufficiently active and active group, respectively.

#### Affective responses

The Feeling Scale (FS) [28] is an 11-point bipolar scale ranging from +5 to -5, commonly used to measure affective response (pleasure/displeasure) during exercise. This scale presents the following verbal anchors: -5 = very bad; -3 = bad; -1 = fairly bad; 0 = neutral; +1 fairly good; +3 = good; and +5 = very good. Previous studies recommended this scale to measure affective responses during exercise [16,19–21,28]. The subjects received standard instructions regarding to the use of the FS in the initial screening, before the maximal exercise test, and before the HIIE bout, according to Hardy and Rejeski [28]: “while participating in exercise it is quite common to experience changes in mood. Some individuals find exercise pleasurable, whereas others find it to be unpleasurable. Additionally, feeling may fluctuate across the time. That is, one might feel good and bad a number of times during exercise. Scientists have developed a scale to measure such responses. [At this point subjects were presented with a copy of the FS]”. FS values were recorded during the last 10s of each work bout during the HIIE session.

#### Rating of perceived exertion

The whole-body perceived exertion during the HIIE bout was assessed using the Borg’s RPE (6–20) Scale. Before the maximal exercise test, the meaning of perceived exertion was explained to the subjects. Perceived exertion was defined as the subjective intensity of effort, strain, and/or fatigue that the subjects can feel during exercise [29]. The low and high perceptual anchors for the Borg’s RPE scale were established during the maximal exercise test. A rating of 6 (low anchor, “very, very light”) was assigned to the lowest exercise intensity, while a rating of 20 (high anchor, “very, very hard”) was assigned to the highest exercise intensity. RPE values were recorded during the last 10s of each minute throughout the maximal exercise test and the HIIE work bouts.

#### Low-volume HIIE protocol

Previous to the HIIE bout, subjects performed a warm-up for 5-min at 50% of MTV. The HIIE consisted of 10 sets of 60s work bouts at 90% of MTV interspersed with 60s of active recovery at 30% of MTV. This practical low-volume HIIE model was adapted from Gibala et al. [12] and was chosen because it has been suggested as a feasible and tolerable exercise prescription for both healthy and clinical populations. The HIIE bout, including the warm-up and cool-down was completed in 30 minutes. In the last 10s of each work bout, the subjects reported their perceived exertion (Borg’s RPE Scale, 6–20) and the affective response (FS, +5 to -5). The order of presentation of the Borg’s RPE Scale and FS was randomized. HR was continuously recorded throughout the HIIE bout (Polar Electro^®^, Oy, Kempele, Finland).

### Statistical analysis

Data are expressed as mean and standard deviation (SD). Normality was tested using the Shapiro-Wilk test. To compare subjects’ characteristics between groups, as well as the mean, highest, and lowest affective response, affective response at the highest RPE, and the mean HR during the HIIE bout, the independent-samples t test was used. Cohen’s d was used to calculate the effect size of these analyses. A two-factor, group (active and insufficiently active) x time (work bouts 1, 2, 3, 4, 5, 6, 7, 8, 9, and 10), mixed-model repeated measures ANOVA analysis was conducted to compare HR, affective responses, and RPE during the HIIE bout. Whenever the sphericity assumption was violated, the degrees of freedom were adjusted and reported using the Greenhouse-Geisser épsilon correction. Partial eta squared (η^2^_p_) was used to determine the effect size of these analyses. If necessary, Tukey’s pos hoc test was used to determine where the significant differences occurred. The chi-squared test was used to verify a possible difference in the distribution of subjects that presented the mean FS score as positive or negative in each group, which were categorized as “unpleasant HIIE” or “pleasant HIIE”; moreover, the frequency of the positive and negative affective responses in the beginning (work bouts 1 to 3), in the middle (work bouts 4 to 7), and in the end (work bouts 8 to 10) of the HIIE bout between the groups was compared using chi-squared test. Pearson product-moment correlation coefficient was used to examine a possible relationship between the affective and RPE responses in both groups. For the analyses, the significance level was set at 5% (p < 0.05). All data were analyzed using SPSS^®^ 20.0 for Windows (SPSS, Inc., Chicago, IL). A post hoc statistical power analysis was conducted using G*Power version 3.1.9.2.

## Results

As expected, the active men displayed a higher performance in the exercise test (maximal treadmill velocity; p < 0.01) and presented higher levels physical activity as compared with the insufficiently active group (p < 0.01; Table 1).

**Table 1:** Subjects’ characteristics and data of the maximal exercise test.

Characteristics	Active (n = 29)	Insufficiently Active (n = 29)
Age (yr)	25.7 ± 3.5	25.0 ± 3.6
Height (cm)	176.0 ± 6.0	175.0 ± 6.0
Weight (kg)	76.7 ± 10.1	76.5 ± 9.8
Body mass index (kg/m^2^)	24.7 ± 2.5	25.1 ± 2.9
Maximal heart rate (bpm)	191.1 ± 11.6	192.4 ± 8.0
Maximal treadmill velocity (km/h)	15.9 ± 1.6*	13.9 ± 1.3
IPAQ score (MET.min.wk^-1^)		
Walking	290 ± 290*	160 ± 100
Moderate physical activity	1034 ± 696*	159 ± 128
Vigorous physical activity	1101 ± 935*	176 ± 142
Total physical activity	2425 ± 1007*	495 ± 88

Note: IPAQ = International Physical Activity Questionnaire.

*Different from the insufficiently active group (p < 0.05). Data expressed as mean ± SD.

**Fig 1:**
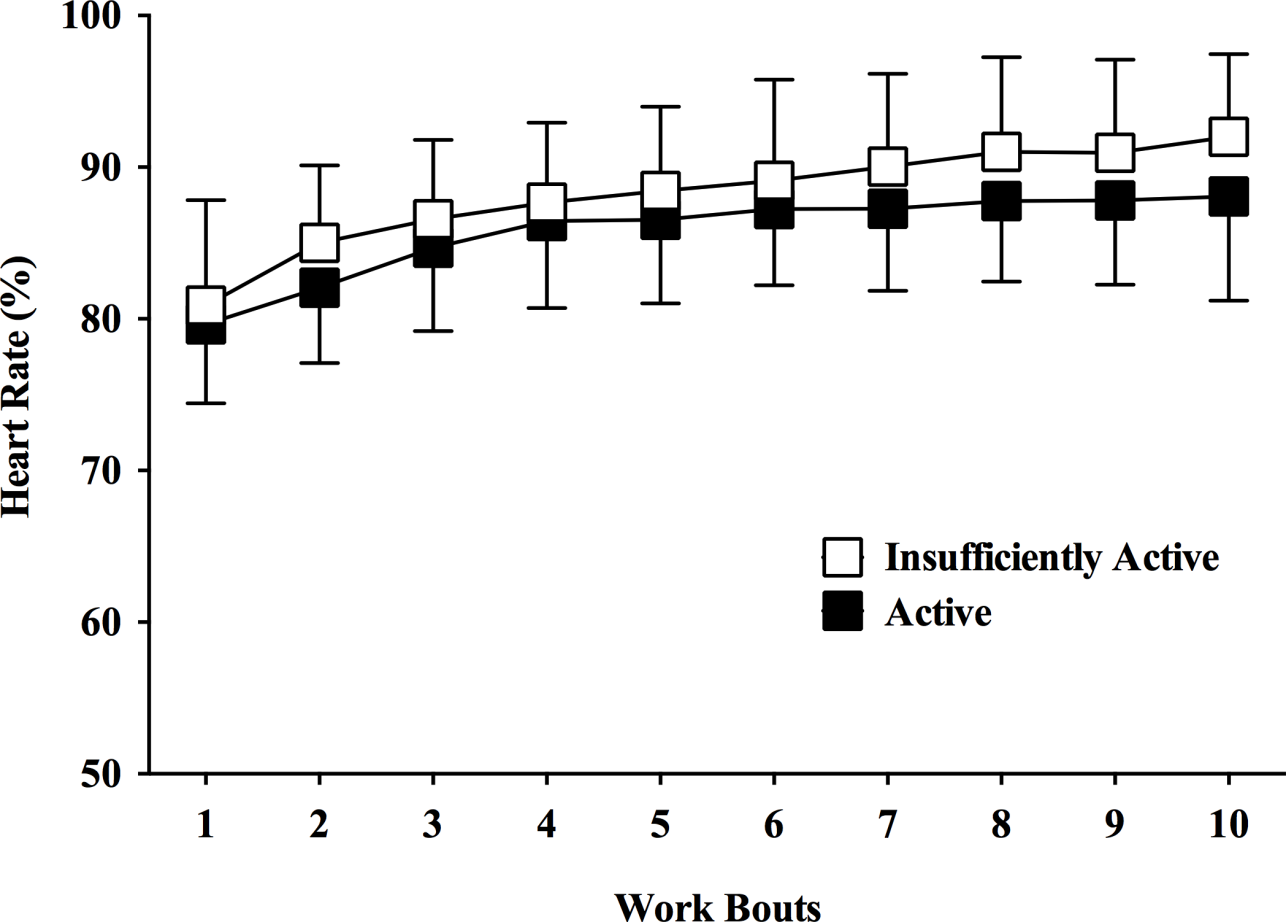
Heart rate responses during a single bout of high-intensity interval exercise in active and insufficiently active men. Data expressed as mean ± SD.

Fig 1 shows the HR responses during the HIIE bout. All participants completed the HIIE bouts. There was only a significant main effect of time [*F*(3.119,174.662) = 83.486, p < 0.001, η2p = 0.599], while no significant interaction group by time [*F*(3.119,174.662) = 1.859, p = 0.136, η2p = 0.032] and no main effect of group [F(1,56) = 3.126, p = 0.083, η2p = 0.053] was observed. Moreover, there was no difference in the mean %HRmax (85.8 ± 5.0% vs. 88.2 ± 5.4%; p = 0.083) between active and insufficiently active groups.

### Affective responses

**Fig 2:**
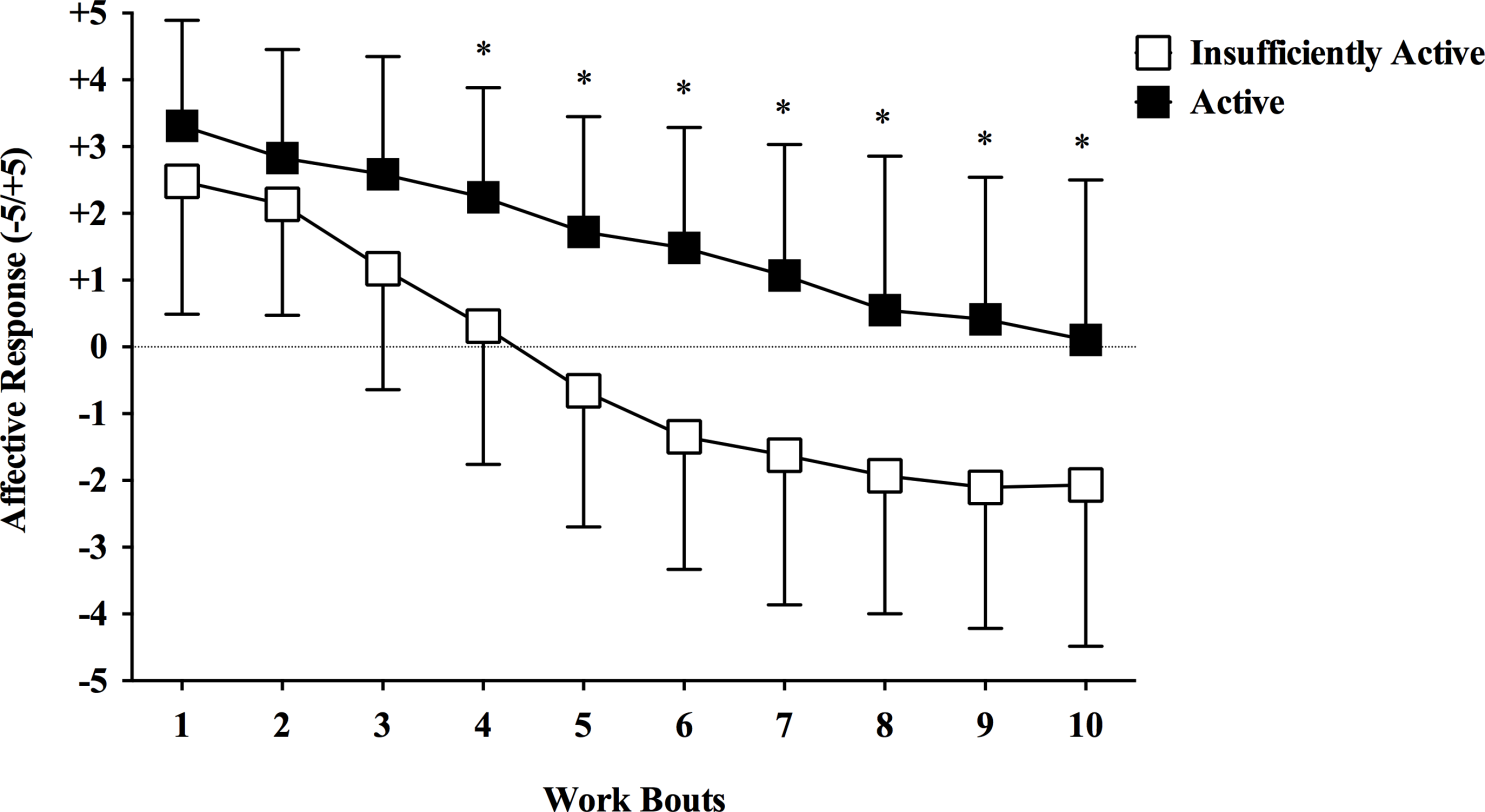
Affective responses during a single bout of high-intensity interval exercise in active and insufficiently active men. *Different from the insufficiently active group (p < 0.05). Data expressed as mean ± SD.

Fig 2 shows the affective responses during the HIIE bout. There was a significant main effect of time [*F*(2.680,150.092) = 95.248, p < 0.001, η2p = 0.630], significant interaction group by time [*F*(2.680,150.092) = 6.897, p < 0.001, η2p = 0.110], and main effect of group [F(1,56) = 20.378, p < 0.001, η2p = 0.267]. Tukey’s pos hoc analysis revealed that the insufficiently active group presented lower affective response from the work bout 4 to 10 (p < 0.001). Also, the insufficiently active group displayed lower values of mean (p < 0.001), lowest (p < 0.001), and highest affective response (p < 0.001), as well as lower values of affective response at the highest RPE (p < 0.001) than the active group (Table 2). Moreover, the insufficiently active group presented a higher distribution of subjects categorized as “unpleasant HIIE” (62.1 vs. 17.2%; p = 0.001) (Table 3) as well as a higher frequency of negative affective responses in the middle (work bouts 4 to 7; p < 0.001) and in the end (work bouts 8 to 10; p < 0.001) of the HIIE bout (Table 4).

**Table 2:** Mean affective response, lowest affective response, highest affective response, and affective response in the highest rating of perceived exertion during a single bout of high-intensity interval exercise in active and insufficiently active men.

Variables	Active (n = 29)	Insufficiently active (n = 29)	ES
Mean affective response	1.6 ± 1.6*	-0.4 ± 1.8	1.2
Lowest affective response	-0.2 ± 2.3*	-2.3 ± 2.0	1.0
Highest affective response	3.5 ± 1.5*	2.7 ± 1.6	0.5
Affective response at the highest RPE	0.1 ± 2.4*	-2.1 ± 2.1	1.0

Note: RPE = rating of perceived exertion; ES = effect size.

*Different from the insufficiently active group (p < 0.01). Data expressed as mean ± SD.

**Table 3:** Categorical analysis of the high-intensity interval exercise bout according to the affective responses in active and insufficiently active men.

	Unpleasant HIIE	Pleasant HIIE	p
**Active**	05 (17.2%)	24 (82.8%)	0.001
**Insufficiently active**	18 (62.1%)	11 (37.9%)	

Note: HIIE = high-intensity interval exercise. For this analysis the mean affective response was used to categorize the high-intensity interval exercise bout as “unpleasant” (negative affective response) or “pleasant” (positive affective response).

**Table 4:** Frequency of the positive/neutral and negative affective responses in the beginning, in the middle, and in the end of the high-intensity interval exercise in insufficiently active and active men.

Work bouts	Positive/Neutral Affect		Negative Affect	
	Insufficiently Active	Active	Insufficiently Active	Active
1–3	79 (91%)	85 (98%)	08 (9%)	02 (2%)
4–7	47 (40%)	99 (85%)*	69 (60%)	17 (15%)*
8–10	19 (22%)	51 (59%)*	68 (78%)	36 (41%)*

Note

*Difference from the insufficiently active group (p < 0.001). Positive/Neutral Affect: values ≥ 0 in the Feeling Scale; Negative Affect = values < 0 in the Feeling Scale.

A post hoc statistical power analysis for the differences in the affective responses between insufficiently active and active groups was conducted to determine the achieved power, based on the investigated sample size (n = 58), an alpha of 0.05, and the achieved effect size. For mixed-model repeated measures ANOVA analysis, the achieved power for the interaction group by time was 98% and the main effect of group was 97%. For independent-samples t test the achieved power was 100%, 100%, 76%, and 100% for the mean, lowest, and highest affective responses, and the affective response at the highest RPE, respectively.

### Rating of perceived exertion

**Fig 3:**
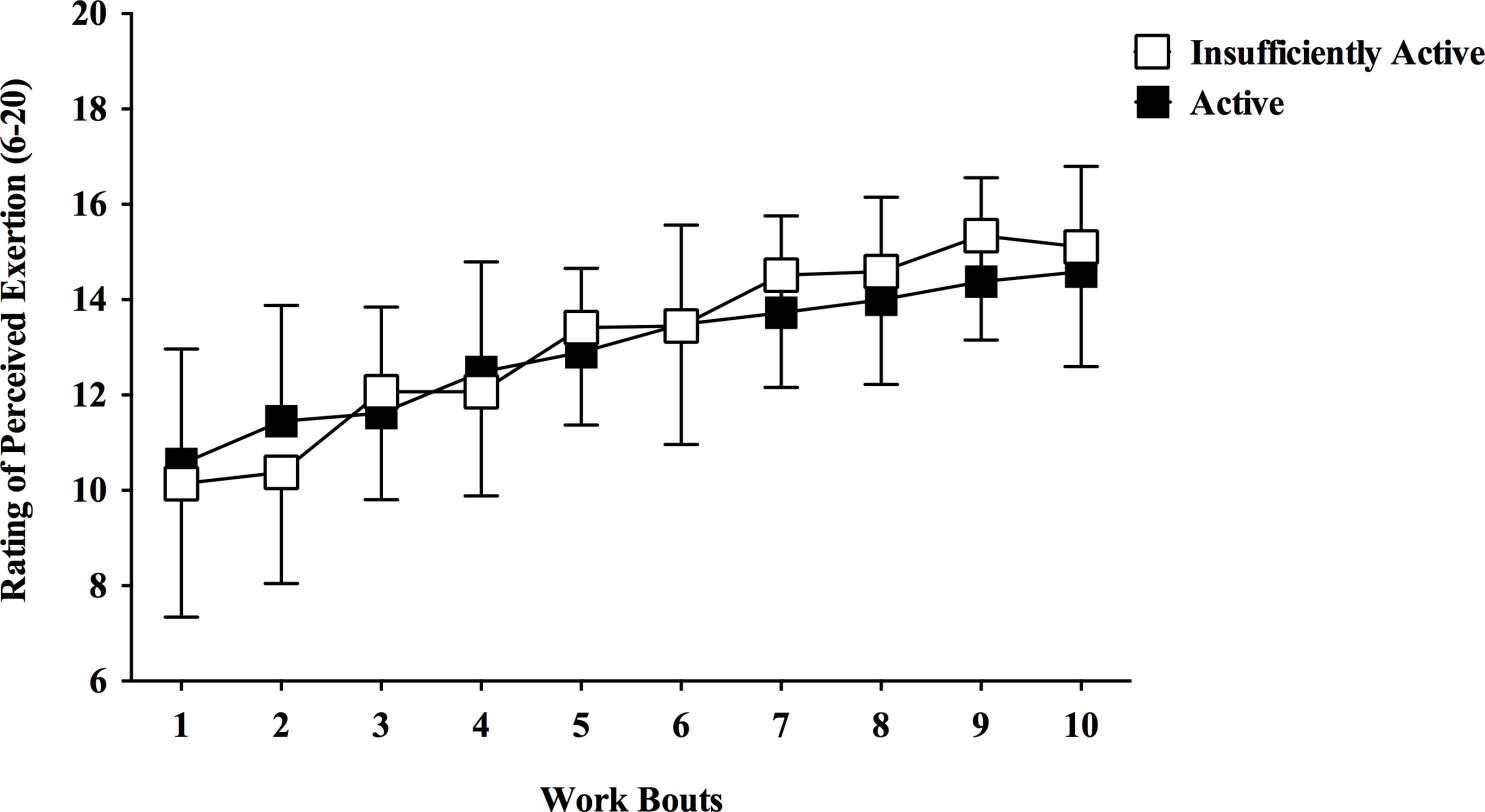
Rating of perceived exertion during a single bout of high-intensity interval exercise in active and insufficiently active men. Data expressed as mean ± SD.

Fig 3 shows the RPE responses during the HIIE bout. There was a significant main effect of time [*F*(3.135,175.559) = 80.478, p < 0.001, η2p = 0.590] and interaction group by time [*F*(3.135,175.559) = 3.253, p = 0.021, η2p = 0.055]. However, there was no main effect of group [*F*(1,56) = 0.149, p = 0.701, η2p = 0.003].

**Fig 4:**
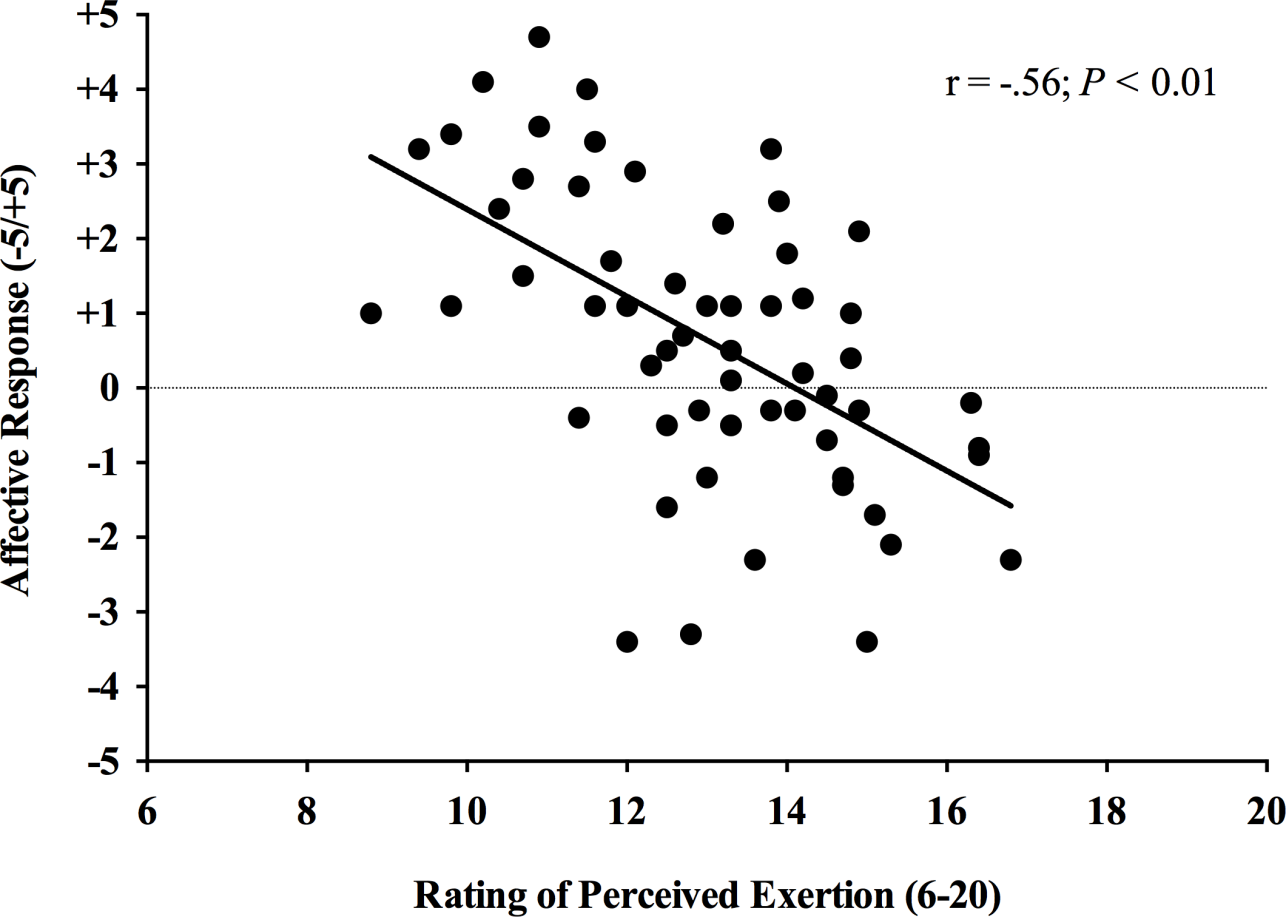
Correlation analysis between affective response and rating of perceived exertion during a single bout of high-intensity interval exercise.

Fig 4 shows the correlation analysis of RPE and affective response during the HIIE bout in the active and insufficiently active groups. There was a negative correlation between RPE and affective response for both groups (p < 0.001; r = -.74 for the active groups and r = -.51 for the insufficiently active group).

## Discussion

Our main finding was that insufficiently active and active men reported similar feelings of pleasure to the first work bouts, while the insufficiently active group displayed negative affective responses over time (from work bout 4 to 10) during a low-volume HIIE protocol, confirming the initial hypothesis. To the best of our knowledge, this is the first report to compare the affective responses to HIIE between subjects with different physical activity status.

During HIIE subjects perform repeated work bouts at high-intensity (close or above respiratory compensation point), which generate a cumulative fatigue and exacerbate the stress imposed to the organism (i.e., internal load) over time. Previous studies demonstrated an increase in VO2, HR, blood lactate concentration, and RPE over the work bouts during different HIIE protocols [30,31]. In our study, HR and RPE increased over the work bouts for both groups (Figs 1 and 3). The opposite pattern was observed for the affective responses, which decreased over the work bouts for both groups (Fig 2 and Table 4). This is partially consistent with the “dual-mode”, which states that there is a negative relationship between exercise intensity and feelings of pleasure, and that exercise intensities above the ventilatory threshold (close to respiratory compensation point) generate homogenous feelings of displeasure [32,33]. Interestingly, only the insufficiently active group reported consistent displeasure during the high-intensity work bouts over time, which was more evident in the second half of the HIIE bout.

Oliveira et al. [34] suggest that a high dependence of the anaerobic metabolism during HIIE negatively influences affective response (i.e., negative feeling of pleasure and high arousal). The authors found that young healthy subjects reported more displeasure during a HIIE bout (~7 work bouts of 2 min at 100% of VO_2_peak interspersed with ~60 s of passive recovery) as compared with an equalized bout of continuous exercise at 85% of the respiratory compensation point (RCP). In our study, it is possible to speculate that the insufficiently active men presented a higher metabolic stress as compared with the active men, as there is an important heterogeneity regarding to the percentage of HRmax equivalent to the individual anaerobic threshold [35]. Myer et al. [36] observed in a moderate to high endurance-trained group that at 85% of HRmax the blood lactate concentration varied from ~1.5 to 5 mmol/L. Moreover, the individual anaerobic threshold of this group varied from 87 to 116% of the workload equivalent to 85% of HRmax. We found a mean HR response during the work bouts of ~86 and 88% of HRmax for the active and insufficiently active groups, respectively. Despite the similar HR responses and the same relative workload (i.e., 90% of MTV), it is possible that the insufficiently active group presented a higher anaerobic contribution during the HIIE protocol, mainly at the last work bouts.

Furthermore, a probable higher dependence of the anaerobic metabolism during the HIIE in the insufficiently active group may have intensified the afferent interoceptive signals from the body to the brain areas related to the generation of the affective response (i.e., prefrontal cortex [PFC] and subcortical areas). The “dual-mode” model states that at high-intensity effort (i.e., above the VT), the functional capacity of the PFC becomes challenged by the intensified interoceptive cues. This induces a deregulation of the PFC, resulting in a negative-affective response, mainly driven by subcortical areas [32,37,38]. Tempest et al. [37] confirmed the limited functional capacity of the PFC at RCP at the point of exhaustion during an incremental test in healthy individuals accompanied by a high displeasure response. Despite the differences between an incremental test and a HIIE bout, the findings of Tempest et al. [37] may, at least in part, explain our results, considering that the anaerobic contribution was higher in the insufficiently active group during the HIIE protocol.

Another important aspect that may explain the difference in the affective response between the groups is the tolerance to exercise intensity, which is defined as a trait that influences one’s ability to continue exercising at levels of intensity associated with discomfort or displeasure [39]. Recently, Tempest et al. [40] found that individuals who self-reported less tolerance to exercise intensity presented more displeasure at RCP and at exhaustion during a maximal incremental test. Previous studies revealed that physical activity participation [41] and VO2max [42] is associated with tolerance to exercise intensity; i.e., less active subjects present lower tolerance to exercise intensity. Considering the lower participation in physical activity and the lower MTV reached during the exercise test (a marker of cardiorespiratory fitness), it is probable that the insufficiently active group has a lower tolerance of exercise intensity. Furthermore, the insufficiently active group may present a lower anaerobic and buffering capacity above the VT. It is possible that these aspects influenced for the negative affective responses reported by this group, mainly during the last work bouts when the contribution of the anaerobic metabolism is higher compared to the first work bouts.

It is possible to think that high attentional associative thoughts and poor sense of self-efficacy may also be involved in the displeasure felt by the insufficiently active group over time [33]. However, we observed that some subjects from the insufficiently active group perceived the HIIE bout as pleasant (~17%) and some individuals from the active group perceived the HIIE bout as unpleasant (~38%) (Table 3). Therefore, the subjects’ exercise preference or personality factors may be associated with this considerable heterogeneity of the affective responses during a HIIE bout. In this sense, it is important to analyze which psychological aspects may be associated with the affective responses to HIIE. Further investigations would do well to collect further data on exercise preferences, exercise motives and personality.

Moreover, some personal characteristics such as prior exercise experience and familiarity with the mode of exercise could substantially influence the cognitive processes involved in the generation of the affective responses to exercise [21,43]. Thus, inexperience with HIIT can lead individuals to experience less positive affective responses. Therefore, the results of the present study suggest that even within a group of regular exercisers, prior exercise experience and familiarity with the mode and/or protocol of exercise may significantly influence the affective responses to a HIIE bout. However, the reasons for this phenomenon remain unclear.

Despite the lower affective response reported by the insufficiently active men, we found a similar RPE between groups. The same finding was previously observed during continuous exercise protocols, mainly at high-intensities [18,19,44]. It should be noted that perceived exertion and the affective valence are not isomorphic constructs. In particular, while the former describes “what” a person feels, the latter emphasizes “how” a person feels [28]. Thus, less active subjects seem to interpret the hard workloads more negatively during continuous and during HIIE. Another interesting finding of our study was the significant negative correlation between RPE and the affective response, independent of physical activity status (Fig 4). In this sense, Oliveira et al. [45] found that RPE, but not HR or VO2, predicted the affective response during continuous and HIIE. These authors suggest that the pattern of affective response seems to be modulated not only by the intensity of exercise, but mostly by “how” the individuals perceive this intensity. These findings support our results, as the subjects who reported higher values of RPE presented lower FS values.

To date, few studies investigated the affective responses to a HIIE bout and the results are contradictory [30,34,46–51]. Oliveira et al. [34] found that young healthy individuals reported displeasure during a HIIE protocol, especially after the half of the bout (quintile 3: -0.27 ± 2.86; quintile 4: -2.17 ± 2.49; quintile 5: -2.67 ± 2.64). However, the practical application of this HIIE protocol may be limited given that 50% of the participants (8 of 15 subjects) were unable to finish the task. Wood et al. [30] found similar affective responses between HIIT (8 bouts of 60s at 85% Wmax with a 60s active recovery at 25% Wmax) and SIT (8 of 30s at 130% Wmax with a 90s active recovery at 25% Wmax) in physically active subjects. In both protocols the subjects reported a negative affective response in the last work bout (HIIE: -1 ± 2.4; SIT: -2 ± 2.5). Similarly, Saanijoki et al. [47] observed that sedentary middle-age men reported consistent displeasure during an ‘all-out’ HIIE protocol (4–6 x 30s ‘all-out’ effort at ~180% of VO_2_max interspersed with 4 min of recovery). Interestingly, the negative affective responses were attenuated over two weeks of training (six HIIE bouts).

On the other hand, Jung et al. [47] reported that insufficiently active men and women decreased their affective responses over time during a single bout of low-volume HIIE (10 x 60s at ~90% of HRmax with 60 s of active recovery), but not in an unpleasant way (2.5 and 0.4 in the beginning and in the end of the bout, respectively). Kilpatrick et al. [49], Martinez et al. [50] and Astorino et al. [51] found similar results regarding the decreased affective responses over time during low-volume HIIE protocols. However, these authors found higher positive affective responses during low-volume HIIE protocols (~2–3 on FS) in moderately fit [49], overweight/obese insufficiently active subjects [50], and sedentary young women [51] using work bouts between 30 and 60s, intensities at or above ventilatory threshold (VT), and a ratio of 1:1 (work bout/recovery). Martinez et al. [50] also found that a HIIE protocol with longer work bouts (i.e., 120s) was perceived as less pleasurable (0.2 ± 2.8 on FS) by the overweight/obese insufficiently active subjects.

Thus, it seems to be clear that the first work bouts of low-volume HIIE protocols at intensities between 80–100% of HRmax with shorter durations (30-60s) may be perceived in a pleasant way for active and insufficiently active subjects. Despite this, perceived confidence to engage in HIIT in a lab setting with supervision and encouragement from exercise physiologists does not necessarily translate very well into confidence to undertake such exercise independently. Moreover, the utilization of the HIIT during unsupervised setting, in which the onus is placed on inexperienced sedentary and/or low active individuals to self-select the appropriate exercise intensity, is likely to be problematic. In a previous study, Lunt et al. [52] evidenced that the improvement in cardiorespiratory fitness in a cohort of overweight/obese subjects undertaking HIIT in a ‘real world’ setting was modest compared to moderate intensity continuous exercise. The main reason for this finding relates to reduced adherence to the HIIT program. Thus, further research is needed to explore whether the HIIT can be successfully implemented and maintained in a real life setting for less active and unfamiliar subjects with supra-threshold exercise intensity. It is important to highlight that the acceptability and feasibility of HIIT is in its infancy and further research is necessary, mainly in a real world setting and over an extended period of follow-up.

In a public health perspective, it is important to highlight that pleasant exercise can improve adoption and adherence to prescribed exercise programs, and may promote future exercise behavior [53]. Thus, it is important that professionals consider the affective responses during an exercise bout, while the long-term adherence to exercise is a recurrent challenge [54]. Therefore, we reinforce that the feelings of pleasure experienced during acute bouts of exercise become an important aspect of exercise prescription and monitoring. In this sense, we suggest that HIIE should be used with caution for beginners and less active individuals during physical activity programs.

Some strengths and limitations of this study are warrant to mention. First, this study did not measure the physical activity status of the participants directly; we used a questionnaire, which may under or overestimate the current subjects’ physical activity level, although previous studies have shown acceptable validity [24,25,55,56]. Second, we did not measure the cardiorespiratory fitness by gas analysis. Although the groups presented different cardiorespiratory fitness based on their results from maximal exercise test (i.e., maximal treadmill velocity), the assumption that low physical activity level associated with low cardiorespiratory fitness influences the affective response during HIIE requires further investigation. Third, we only included non-obese young healthy males in this study. Therefore, our findings may not be directly transferable to other populations or to females. Fourth, the mental status of the participants (e.g., mood, stress, depression, etc.) was not assessed in the initial screening and prior to the HIIE bout. Despite the above mentioned limitations, it is important to highlight some aspects that favor the ecological validity of this study: i) application of a low-volume HIIE protocol to daily exercise; ii) utilization of the treadmill, commonly used in exercise facilities rather than Wingate tests on specialized cycle ergometer; iii) use of simple tools to assess psychometric responses to exercise (i.e., Borg’s RPE scale and Feeling Scale), which may be easily integrated into practice by exercise professionals.

## Conclusions

Overall, insufficiently active and active subjects report feelings of pleasure to the first few work bouts (i.e., 3–4) during low-volume HIIE, while the affective responses become more unpleasant over time for insufficiently active subjects. Despite the physiological benefits of current low-volume HIIT protocols (i.e., 10 x 60s at ~90% of HRmax with 60s recovery) in improving health status and fitness, it is important to consider that this protocol is likely to be experienced as unpleasant for less active subjects, especially in the last work bouts. Thus, considering the impact of feeling states during exercise for future exercise participation and adherence, investigations on the effects of HIIT protocols including a fewer number of work bouts on health status and fitness of less active subjects would be interesting, especially in the first training weeks. Moreover, further research is needed to examine adherence to HIIT protocols and to explore whether this exercise modality can be successfully implemented and maintained in a real life setting with those less physically active and unfamiliar with vigorous intensity exercise.

## Supporting Information

S1 File Data set. Characteristics of the sample and individual responses to high-intensity interval exercise bout.
(XLSX)
