# Correction: Feeling of Pleasure to High-Intensity Interval Exercise Is Dependent of the Number of Work Bouts and Physical Activity Status

**DOI:** 10.1371/journal.pone.0153986

**Published:** 2016-04-14

**Authors:** Danniel Thiago Frazão, Luiz Fernando de Farias Junior, Teresa Cristina Batista Dantas, Kleverton Krinski, Hassan Mohamed Elsangedy, Jonato Prestes, Sarah J. Hardcastle, Eduardo Caldas Costa

## Notice of Republication

S1 File was published in error. The error was corrected in the HTML version of this article on April 6, 2016, and the corrected S1 File is available in the Supporting Information of this article.

## Supporting Information

S1 File Data set. Characteristics of the sample and individual responses to high-intensity interval exercise bout.
(XLSX)

## References

[1] Frazão DT, de Farias Junior LF, Dantas TCB, Krinski K, Elsangedy HM, Prestes J, et al (2016) Feeling of Pleasure to High-Intensity Interval Exercise Is Dependent of the Number of Work Bouts and Physical Activity Status. PLoS ONE 11(3): e0152752 doi:10.1371/journal.pone.0152752 27028191 10.1371/journal.pone.0152752 PMC4814045

